# Insulin Resistance in Alzheimer's Disease

**DOI:** 10.3389/fnins.2018.00830

**Published:** 2018-11-13

**Authors:** Laís S. S. Ferreira, Caroline S. Fernandes, Marcelo N. N. Vieira, Fernanda G. De Felice

**Affiliations:** ^1^Institute of Medical Biochemistry Leopoldo de Meis, Federal University of Rio de Janeiro, Rio de Janeiro, Brazil; ^2^Institute of Biomedical Sciences, Federal University of Rio de Janeiro, Rio de Janeiro, Brazil; ^3^Institute of Biophysics Carlos Chagas Filho, Federal University of Rio de Janeiro, Rio de Janeiro, Brazil; ^4^Department of Biomedical and Molecular Sciences, Centre for Neuroscience Studies, Queen's University, Kingston, ON, Canada

**Keywords:** insulin signaling, insulin resistance, Alzheimer's disease, obesity, type 2 diabetes, metabolic dysregulation, blood-brain barrier

## Abstract

The epidemiological connection between diabetes, obesity, and dementia represents an important public health challenge but also an opportunity to further understand these conditions. The key intersection among the three diseases is insulin resistance, which has been classically described to occur in peripheral tissues in diabetes and obesity and has recently been shown to develop in Alzheimer's disease (AD) brains. Here we review encouraging preclinical and clinical data indicating the potential of targeting impaired insulin signaling with antidiabetic drugs to treat dementia. We further discuss biological mechanisms through which peripheral metabolic dysregulation may lead to brain malfunction, providing possible explanations for the connection between diabetes, obesity, and AD. Finally, we briefly discuss how lifelong allostatic load may interact with aging to increase the risk of dementia in late life.

## Introduction

Alzheimer's disease (AD), type 2 diabetes (T2D), and obesity are among the most expensive and disabling disorders worldwide. For a long time, the correlation between cognitive impairment and metabolic diseases was undetected. Now, increasingly epidemiological evidence supports an important association among these conditions (Razay et al., 2006; Baker et al., 2011; Crane et al., 2013; Ikram et al., 2017). Accordingly, experimental observations are identifying that markers of metabolic dysregulation are also present in AD, the most remarkable being insulin resistance (Talbot et al., 2012; De Felice, 2013; Boles et al., 2017). However, the molecular mechanisms underlying this crosstalk are still elusive, as well as how central and peripheral insulin signaling operate in AD (Biessels and Despa, 2018).

Over the last decade, cumulative data have reinforced that the brain is an insulin-sensitive organ. The insulin receptor (IR) and related insulin-like growth factor receptors 1 and 2 (IGF1-R/IGF2-R) are expressed not only in rodent hypothalamus, the critical brain region for metabolic control, but also in cortex, hippocampus, thalamus, olfactory bulb, and in lower levels, in cerebellum, striatum, midbrain, and brainstem (Fernandez and Torres-Alemán, 2012; Kleinridders et al., 2014).

Beyond the canonical role of insulin in whole body metabolism regulation (Brüning et al., 2000), insulin also modifies neuronal activity promoting synaptic plasticity (Wan et al., 1997; Schmitz et al., 2018) and improves memory function in mammalian brain (Park et al., 2000; Benedict et al., 2007). Glial insulin signaling functions are gaining attention. Hypothalamic astrocyte IRs control glucose-induced activation of POMC neurons, central and peripheral response to glucose availability, and glucose transport through the blood-brain barrier (BBB, García-Cáceres et al., 2016). Additionally, insulin modulates proinflammatory cytokine secretion in microglia and astrocytes *in vitro* (Spielman et al., 2015; Kurochkin et al., 2018). Under brain injury, IGF1 can be produced by activated microglia and is required for vessel remodeling (Walter et al., 1997; Lopez-Lopez et al., 2004). Further studies are required to decipher the complex regulation of insulin signaling in distinct cell types, but these data suggest that IR/IGF-Rs play a role in central nervous system (CNS) physiology.

Remarkably, several studies show that insulin signaling is impaired in the brains of AD patients and AD experimental models (Boyt et al., 2000; Craft et al., 2003; Steen et al., 2005; Bomfim et al., 2012; Hiltunen et al., 2012; Talbot et al., 2012). Neuronal insulin resistance can be induced by Aβ-oligomers in primary cultures of hippocampal neurons and by intracerebroventricular injection of AβOs in mice and monkeys. It is mediated by TNF-α activation and IRS inhibition and has major impact on synaptic dysfunction, impaired synaptic plasticity, and synapse loss (Townsend et al., 2007; De Felice et al., 2009; Bomfim et al., 2012; Batista et al., 2018) Remarkably, our group found that icv injection of Aβ oligomers also induce peripheral glucose intolerance with classic hallmarks of peripheral insulin resistance, a process also observed in transgenic AD mice models(Clarke et al., 2015) and that may underlie increased risk for diabetes in AD (Janson et al., 2004). Additionally, anti-diabetic drugs exerts beneficial effects on cognition, synapse protection, insulin signaling deficits, and other AD-related pathological mechanisms, such as endoplasmic reticulum stress and chronic inflammation (Lourenco et al., 2013; Sebastião et al., 2014; Batista et al., 2018; Tai et al., 2018).

Here, we review evidence regarding similar mechanisms of insulin resistance shared by T2D, obesity, and AD. We will discuss recent findings that help explain the connection between peripheral metabolic deregulation and AD, the cause-consequence between these diseases and how boosting insulin pathway provide therapeutic alternatives for treating AD.

## Crosstalk between peripheral metabolic diseases and brain pathology

A key question to understand the connection between diabetes and obesity to AD is to learn how metabolic impairment in periphery may cause pathological alterations in the brain.

Fatty acids and lipids in general are well-established players in obesity, diabetes, and AD. A recent meta-analysis study revealed the potential mechanistic, diagnostic, and therapeutic implications of different classes of lipids in AD (Zarrouk et al., 2017). Obesity and diabetes present inflammatory components (Wang et al., 2018). Indeed, obese patients often display basal low-grade systemic inflammation in adipose tissue and increased susceptibility to immune-mediated diseases (Sun et al., 2012; Mraz and Haluzik, 2014). Free fatty acids (FFA) from feeding trigger an inflammatory cascade initiated by Toll-like receptor 4 (TLR4) stimulation, releasing pro-inflammatory cytokines such as TNF-α and interleukins IL-1β and IL-6 (Weisberg et al., 2003; Shu et al., 2012). High levels of FFA can further inhibit the anti-lipolytic action of insulin, increasing the rate of FFA release into the bloodstream (Guenther, 2009). FFA binding to BBB endothelial cell alters permeability, allowing FFA infiltration into the brain (Rapoport, 2001). Hence, the increase in cerebral FFA triggers detrimental events such as ceramide production, pattern recognition receptors activation, inflammation, and ER stress (Spiegel, 2005; Guenther, 2009; Groop et al., 2018). When the brain detects homeostatic disruption, microglia are activated leading to neuroinflammation (Jha et al., 2016). Saturated fatty acids and mono-unsaturated fatty acids were shown to activate microglial NF-kB pathway in a TLR4-dependent manner, leading to increased production of proinflammatory cytokines, and reactive oxygen species (ROS, Wang et al., 2012; Arnold et al., 2014; Button et al., 2014; Carroll et al., 2018). Saturated fatty acids were also shown to induce TLR4-dependent activation of astrocytes in culture leading to cytokine production (Gupta et al., 2012; Wang et al., 2012).

Advanced glycation end-products (AGEs) also represent a common feature in diabetes and AD, and may be involved in the crosstalk between periphery and CNS that underlies this connection. AGEs are the product of unspecific and uncontrolled reactions between proteins or lipids with sugars. AGEs increase during normal aging, but their formation is promoted in glucose-rich environments, such as in hyperglycemia. Interestingly, elevated AGEs levels are also observed in AD brains (Shuvaev et al., 2001; Choei et al., 2004; Takeuchi et al., 2007). Importantly, AGE-receptor (RAGE) was suggested as a possible receptor for Aβ (Yan et al., 1996) and to mediate Abeta toxic mechanisms such as induction of ER-stress (Chen et al., 2018). Moreover, RAGEs have been shown to promote Aβ production, tau hyperphosphorylation and tangle formation, synaptic impairment cognitive decline, and neurodegeneration (Cai et al., 2016). Downregulating RAGE signaling specifically in microglia prevented synaptic deficit and cognitive impairment and diminished the activation of stress-related kinases in a mouse model of AD (Criscuolo et al., 2017). Importantly, recent evidences indicate that RAGE mediates Abeta impact on BBB integrity and tight junction regulation as well APOE4-induced BBB anomalies (Park et al., 2014; Wan et al., 2014, 2015; Alata et al., 2015).

Collectively, these observations indicate that lipidemic dysregulation in metabolic disorders may lead to BBB permeabilization to FFAs, triggering a cascade of events leading to activation of glial cells, and neuroinflammation. In parallel, increased AGE levels in hyperglycemia may contribute to RAGE-mediated disruption of BBB integrity and further promote brain pathology leading to AD. Proinflammatory cytokines reaching the brain through compromised BBB form a toxic environment for neurons, leading to neuronal insulin resistance, and synaptic dysfunction (Bomfim et al., 2012; Gupta et al., 2012; Lourenco et al., 2013; Kiernan et al., 2016; Vieira et al., 2017, Figure 1). Consistent with this hypothesis, it has been shown that HFD activates inflammatory responses in the mouse hippocampus (Lu et al., 2011; Almeida-Suhett et al., 2017) and impairs insulin signaling (Arnold et al., 2014) whereas a high-AGE diet aggravates AD-like phenotypes in a mouse model of AD.

**Figure 1:**
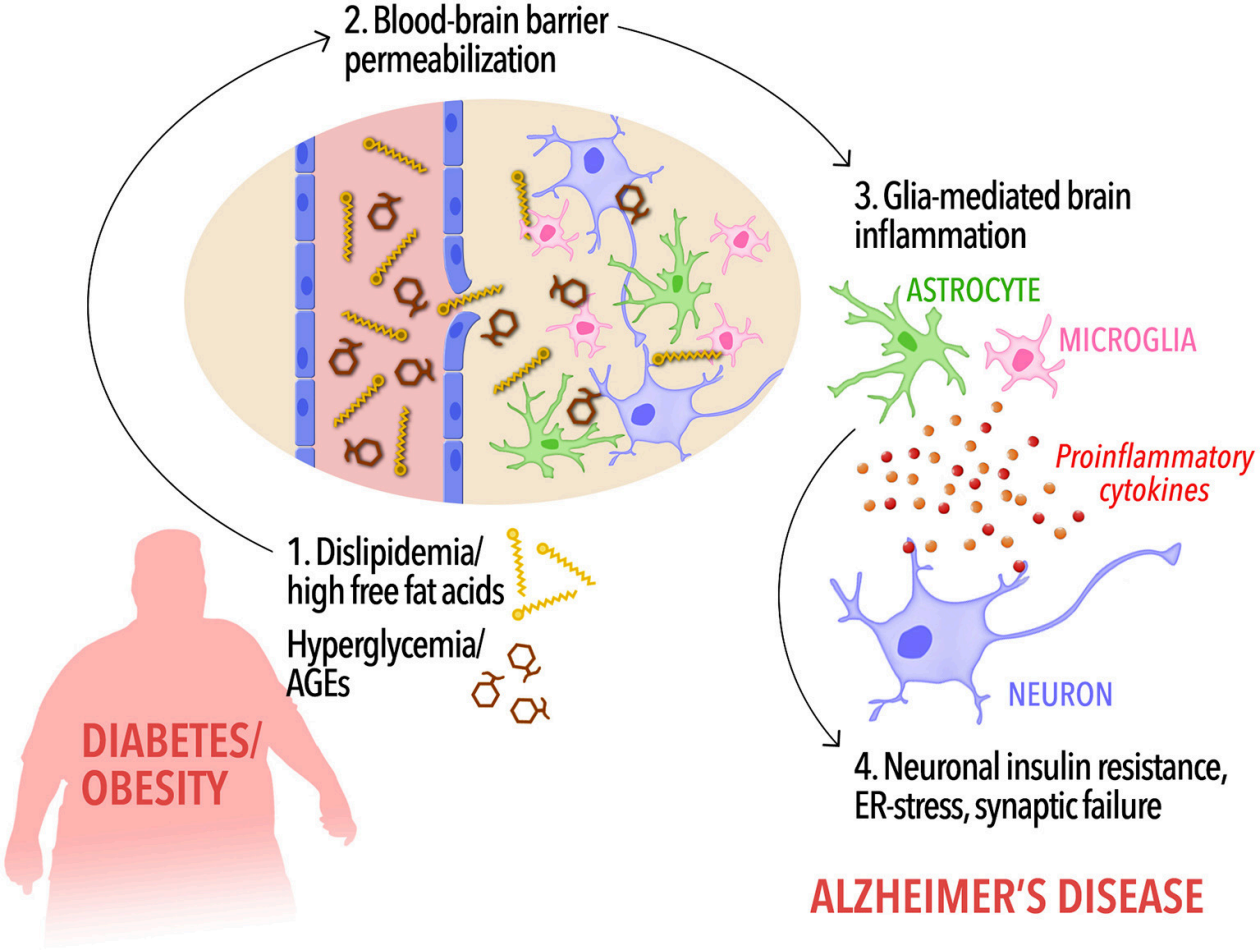
A possible cascade of events connecting peripheral metabolic dysregulation to dementia. In diabetic and/or obese subjects, dyslipidemia, and increased circulating free fat acids as well as hyperglycemia and elevated peripheral AGEs levels (1) may increase blood-brain barrier permeability, allowing the influx of FFAs into the brain (2). Disrupted BBB along with high levels of brain FFAs and AGEs, in turn, would cause activation of microglia and astrocytes and the release of proinflammatory cytokines (3). Low-grade, chronic brain inflammation leads to detrimental events in neurons, including insulin resistance (4), priming the brain to cognitive impairment and Alzheimer's disease.

## The impact of impaired insulin signaling in brain and peripheral cells

Intriguingly, many studies suggest that the incidence of AD is higher in T2D patients and obese individuals, implying common mechanisms driving these disorders (Kivipelto et al., 2005; Razay et al., 2006; Whitmer et al., 2007; Baker et al., 2011; Crane et al., 2013; Ikram et al., 2017). A core feature shared among diabetes, obesity, and AD is insulin resistance (Kullmann et al., 2016). In AD patients brains, impaired insulin signaling is a risk factor but also aggravates the pathology (Matsuzaki et al., 2010; Arnold et al., 2018).

During the past decades, mounting studies correlate the alterations in IR/IGF-R signaling pathways and AD (Boyt et al., 2000; Craft et al., 2003; Bomfim et al., 2012; Hiltunen et al., 2012; Talbot et al., 2012; Pitt et al., 2017). In addition, alterations in other proteins involved in insulin signaling were also described. Insulin-degrading enzyme (IDE) is essential for insulin and Aβ clearance (Farris et al., 2003). Insulin resistance in AD and diabetes can lead to hyperinsulinemia, thereby, saturating IDE for insulin and Aβ degradation. Furthermore, IDE function declines with age, the major risk factor for sporadic AD (Kurochkin et al., 2018). Also, phosphatase and tensin homolog (PTEN), protein kinase B (Akt), and glycogen synthase kinase 3β (GSK3β) are recruited to the synaptic compartments impairing LTP after Aβ exposure (Knafo et al., 2016).

In diabetes and obesity, increased levels of pro-inflammatory cytokines, particularly tumor necrosis factor alpha (TNF-α) activates c-Jun terminal kinase (JNK), resulting in insulin receptor substrate 1 (IRS1) suppression by increasing the inhibitory phosphorylation of Ser^312^IRS1, Ser^636^IRS1, and decreasing activating phosphorylation of Tyr^465^IRS1 (Hotamisligil et al., 1993; Rui et al., 2001; Pedersen et al., 2003). These alterations were also identified in AD pathogenesis: JNK activation and IRS1 inhibition are present in the hippocampus of AD transgenic mice and cynomolgus monkeys after intracerebroventricular (icv) injections of Aβ oligomers (Ma et al., 2009; Forny-Germano et al., 2014). Of note, these alterations in insulin signaling pathway were also described in AD patients. Initial evidence comes from a study showing that AD brains display markedly reduced levels of both insulin and insulin-like growth factors (IGF)-I and –II, accompanied by decreased expression of IR mRNA, IRS-associated PI3K, and activated Akt (Steen et al., 2005). This observations were subsequently corroborated by studies demonstrating progressively increased phosphorylation of IRS-1 in inhibitory serine residues, accompanied by augmented levels of their activated serine-kinases GSK-3, IKK, JNK, mTOR, and PKCζ/λ, as patients evolve from non-demented to MCI to AD (Bomfim et al., 2012; Talbot et al., 2012). Increased IRS-1 serine phosphorylation was later found to co-localize with neurons presenting tau-pathology and tangles (Yarchoan et al., 2014). Additionally, it was observed increased phosphorylation of Ser^312^IRS1 in neural-derived blood exosomes of AD, frontal dementia, and T2D patients. The most interest point of this study is that increased p-Ser^312^IRS1 manifested in prodromal AD patients that sustained these alterations 10 years later, as AD patients (Kapogiannis et al., 2015), suggesting that insulin resistance in AD develops years before clinical manifestations and that neural-derived exosomes carries potential for early AD diagnosis. Exosomal biomarkers for insulin resistance were further associated with morphometric changes in AD brains (Mullins et al., 2017). Notably, the inhibitory phosphorylation of IRS-1 and IRS-2 described in AD patients brains and transgenic mice are correlated to memory deficits and leads to insulin resistant states (Steen et al., 2005; Bomfim et al., 2012; Talbot et al., 2012).

In peripheral tissues, such as the adipose tissue, of diabetes and obesity models, TNF-α release also activates stress kinases IκB kinase (IKK) and double-stranded RNA-dependent protein kinase (PKR), promoting inflammation and inducing endoplasmic reticulum stress besides insulin signaling deregulation (Hotamisligil et al., 1993; Wellen and Hotamisligil, 2005; Yang et al., 2009). Conversely, TNF-α depletion protects mice from obesity-induced insulin resistance (Uysal et al., 1997). Similarly, Aβ oligomers also induce TNF-α secretion and the consequent induction of IKK and PKR activation in AD animal models (Bomfim et al., 2012; Lourenco et al., 2013), suggesting a common mechanism promoting insulin resistance.

Interestingly, icv injections of Aβ oligomers in mice provokes peripheral glucose intolerance, insulin resistance, and inflammation characterized by activation of JNK and IKK and IRS-1 inhibition, connecting AD pathology progression to the development of diabetes (Clarke et al., 2015). In AD patients, it was proposed an association between peripheral and central insulin resistance by correlating [18F]-fln AD patients, i positron emission tomography (FDG-PET) scans in the brain and the homeostasis model assessment of insulin resistance (HOMA-IR) in the blood. The study evaluated AD-vulnerable regions of interest (ROIs) and predicted higher HOMA-IR and lower FDG in all ROIs, indicating that central hypoglycemia and peripheral insulin resistance are related. Additionally, both were associated with immediate and delayed memory deficits in the medial temporal lobe (Willette et al., 2015).

Although not completely elucidated, it is now acknowledged that the alterations of insulin signaling in AD and diabetes are associated. On that account, a great effort has been made to distinguish the selective insulin responses in cellular types and contexts. Most of insulin effects were thought to be mediated by neurons, but recent findings indicate insulin actions on glial cells affecting their functions and whole-body response (Bélanger et al., 2011; García-Cáceres et al., 2016; Fernandez et al., 2017). Astrocytic IR-knockout mice present moderate glucose intolerance and depressive-like behavior. Furthermore, astrocytic-mediated ATP traffic to neurons is impaired, affecting purinergic signaling and, consequently, decreasing dopamine release. Curiously, these phenotypes were only observed in male mice, suggesting a possible sex-specific role for IR in astrocytes (Cai et al., 2018). This could represent a new molecular link between AD and other psychiatric disorders, since clinical evidence has linked depression and AD (Ownby et al., 2006; Modrego, 2010). In line with these observations, knocking out astrocytic IGF1-R in mice debilitates mitochondrial function, elevating ROS production, and impairs working memory. In addition, astrocytic IGF1-R knockout decreases glucose and Aβ uptake by astrocytes, contributing to Aβ accumulation in the brain (Logan et al., 2018).

A neurotoxic astrocyte phenotype induced by activated microglia was recently described. This A1-like astrocyte is associated with aging and aggravation of several neurodegenerative disorders (Liddelow et al., 2017; Clarke et al., 2018). Conversely, targeting microglia-mediated conversion of astrocytes into A1 type with NLYP01, a novel glucagon like peptide 1 receptor (GLP1R) agonist, increases lifespan and reverses the loss of dopaminergic neurons, and behavioral deficits in mouse models of Parkinson's disease (Yun et al., 2018). In the future, it will be interesting to investigate if the beneficial effects of targeting microglia GLP1 pathway offer any improvement to other neurodegenerative diseases, such as AD (Liddelow and Barres, 2017).

The strongest genetic risk factor for late-onset Alzheimer is the isoform ApoE4 of apolipoprotein E (ApoE4) protein, involved in cholesterol metabolism (Strittmatter et al., 1993a,b). Alongside with ApoE4 risk, AD patients also present abnormal levels of 24-hydroxycholesterol (24-OHC), a cholesterol oxide derivative which is important for learning and memory, in the plasma and cerebrospinal fluid (CSF) (Zarrouk et al., 2017). Interestingly, ApoE4 appears to exacerbate AD neuropathology in individuals with T2D (Malek-Ahmadi et al., 2013). It was also observed that insulin resistance in ApoE4 carriers is correlated with higher levels of phosphorylated tau in the CSF (Starks et al., 2015). Increasingly reports are trying to figure out the connection between ApoE4 carriers, development of AD, and metabolic changes associated with obesity and diabetes (Peila et al., 2002; Reiman et al., 2005; Moser and Pike, 2017; Zhao et al., 2017). It was described that ApoE4 genotype aggravates weight gain, impaired glucose metabolism, augmented Aβ plaque load, and gliosis in mice under high fat and sugar diet (Moser and Pike, 2017). Also, human ApoE4 variant interacts with insulin receptor trapping it on endosomes in neurons, reducing IRs availability in neuronal surface and diminishing insulin sensitivity. Remarkably, these effects are age-dependent and accelerated under high fat diet (HFD, Zhao et al., 2017). It was also described that the TREM2-ApoE pathway regulates the switch to neurodegenerative microglia induced by phagocytosis of apoptotic neurons, playing a detrimental role in AD (Krasemann et al., 2017). However, if and how insulin signaling participates in this process remains to be determined. Importantly, TREM2 polymorphisms also determines high risk for late-onset AD, but their role in neurodegenerative diseases are controversial (Jay et al., 2015; Wang et al., 2015). Therefore, the current challenge is to identify distinct roles of insulin signaling in multiple genetic and environmental backgrounds comprising AD risk and development to selectively modulate insulin signaling.

## Beneficial effects of boosting insulin signaling in the context of AD

Drugs currently in use for AD treatment target acetylcholinergic, NMDA-type glutamatergic, and glutaminergic pathways. These therapies offer symptomatic relief of memory defects but do not combat the pathological mechanisms underlying AD progression (Graham et al., 2017). Therapies based on the amyloid hypothesis, aimed to reduce Aβ production, and toxic Aβ aggregates in the brain (Barage and Sonawane, 2015), have proven unsuccessful due to severe collateral effects (Searfoss et al., 2003; Wong et al., 2004), unfavorable pharmacological properties (Vassar, 2014), or fail to improve cognition of AD patients in clinical trials (Salloway et al., 2014; Lasser et al., 2015; Kennedy et al., 2016; Egan et al., 2018; Honig et al., 2018). In this context, it is important to explore alternative targets that may provide symptom relief and ameliorate pathology with minor collateral effects.

On this matter, mounting data indicate that anti-diabetes drugs could be neuroprotective in AD models and clinical studies. Our group has contributed investigating the modulation of insulin signaling by clinically used anti-diabetic drugs in diverse AD models. We identified that insulin prevents Aβ oligomers-induced synapse loss and surface IR reduction *in vitro* (De Felice et al., 2009) and also rescued the PKR-mediated endoplasmic reticulum stress in AD models (Lourenco et al., 2013). Also, we investigated the beneficial effects of GLP1-R agonists, such as exendin-4 and liraglutide, in hippocampal cultures exposed to Aβ oligomers, transgenic AD mice, and cynomolgus monkeys injected icv with Aβ oligomers. These drugs activate insulin-related pathways through G-protein dependent signaling, regardless of IR, and IGF-R (Andersen et al., 2018). Exendin-4 acted decreasing the inhibitory phosphorylation of Ser^312^IRS1, Ser^636^IRS1, and of JNK, while restoring activating Tyr^465^IRS1 phosphorylation, then counteracting insulin signaling impairment, memory deficits and diminishing amyloid plaque load in APP/PS1 transgenic AD mice (Bomfim et al., 2012). Liraglutide reduced tau phosphorylation and prevented IR reduction and synapse loss in a c-AMP dependent manner in cynomolgus monkeys injected icv with Aβ oligomers (Batista et al., 2018). Other groups also showed that liraglutide can reduce inflammation and enhance LTP in AD transgenic mice (McClean et al., 2011; McClean and Hölscher, 2014), that exendin-4 prevents neuronal excitotoxicity in a neurodegenerative rat model (Perry, 2002) and icv insulin injections enhance cognitive performance (Park et al., 2000, Table 1).

**Table 1:** Summary of preclinical and clinical studies on the efficacy of anti-diabetic, insulin-sensitizing drugs on multiple aspects of AD pathology in human patients and animal models.

**Compound**	**Finding**	**Model**	**References**
Insulin	Prevention of AβO induced synapse loss and IR reduction; amelioration of PKR-mediated ER stress	Rat hippocampal neuronal cultures	De Felice et al., 2009; Lourenco et al., 2013.
Insulin	AD patients that are not ε4 carriers have reduced sensitivity to insulin, affecting cognitive performance	AD patients homozygous or not for the apoE-ε4 allele and normal subjects intravenously injected	Craft et al., 2003.
Insulin	Improve verbal memory in MCI AD ε4- subjects after acute insulin administration but not in ε4 carriers	AD patients homozygous or not for the apoE-ε4 allele, mild cognitive impaired patients and normal subjects intranasally administrated	Reger et al., 2006, 2008a
Insulin	Chronic intranasal insulin doses enhanced selective attention, retention of new information and functional status of MCI and early AD subjects	AD patients, mild cognitive impaired patients and normal subjects intranasally administrated	Reger et al., 2008b
Insulin	Only women presented improved working memory after treatment	Healthy men and woman intranasally administrated	Benedict et al., 2008
Liraglutide	Reduction of tau phosphorylation; prevention of IR reduction and synapse loss in a c-AMP dependent manner	Cynomolgus monkeys injected icv with AβO	Batista et al., 2018
Liraglutide	Improvement of memory deficits in novel object recognition test and fear conditioning	Swiss mice injected icv with AβO	Batista et al., 2018
Liraglutide	Restore memory deficits in object regonition test and morris water maze; enhance LTP; Reduce microglial activation; diminish amyloid plaque load	APP/PS1 mice	McClean et al., 2011; McClean and Hölscher, 2014
Exendin-4	Decrease of the inhibitory phosphorylation of Ser^312^IRS1, Ser^636^IRS1 and of JNK, while restoring activating Tyr^465^IRS1 phosphorylation	Rat hippocampal neural cultures	Bomfim et al., 2012.
Exendin-4	Improvement of spatial memory in moris water maze; reduced amyloid plaque load	APP/PS1 mice	Bomfim et al., 2012
Exendin-4 Liraglutide	eIF2α phosphorylation reduction	Rat hippocampal neural cultures, APP/PS1 mice, cynomolgus monkeys injected icv with AβO	Lourenco et al., 2013
GLP-1 Exendin-4	Reduction of neural excitotoxicity	Rat hippocampal neural cultures; Rats injected on the basal nucleus with ibotenic acid	Perry, 2002
Rosiglitazone	Reversal of memory deficits in object recognition test and morris water maze; Aβ levels reduction	AD transgenic mice J20 line	Escribano et al., 2010

Observations taken from preclinical and epidemiological data encouraged clinical trials repurposing insulin as a prospect treatment to AD patients (Table 1). Anti-diabetics exerts neuroprotective effects by mitigating Aβ toxicity, reducing inflammation, and improving memory deficits (Bomfim et al., 2012; Lourenco et al., 2013). In this context, anti-diabetic therapy offers a multitarget approach covering several aspects of AD pathology progression. However, concerns about off-targeting consequences have been raised, as insulin signaling systemically controls various cellular processes. To bypass this problem, one propitious solution is the intranasal delivery, restricting it to CNS and avoiding major peripheral effects such as hypoglicemia, besides being more effective than oral administration (Born et al., 2002; Spetter and Hallschmid, 2015; Schmid et al., 2018). In mild cognitive impairment (MCI) and early AD patients, acute intranasal insulin administration facilitates verbal memory recall. Nevertheless, ApoE4 carriers presented poorer verbal memory recall after the treatment, suggesting a role for ApoE4 genotype in central effects of insulin (Craft et al., 2003; Reger et al., 2006, 2008a). The same group described that chronic intranasal insulin doses enhanced selective attention, retention of new information, and functional status of MCI and early AD subjects (Reger et al., 2008b). Importantly, selective effects of intranasal insulin treatment were observed. It was reported that women show better scores of cognitive improvement than men after the intranasal insulin treatment (Benedict et al., 2008). Conversely, in obese individuals, intranasal insulin have no effect on body weight but improved declarative memory and mood (Schneider et al., 2014). At this moment, there are ten ongoing unpublished clinical trials accessing intranasal insulin beneficial effects on AD patients (NCT00581867, NCT02010476, NCT01436045, NCT01636596, NCT03038282, NCT01767909, NCT00018382, NCT01595646, NCT01547169, NCT02462161).

Besides insulin, GLP1-R agonists are also an alternative to be invested on clinical trials, considering prior positive results in preclinical models (Duarte et al., 2013; Tramutola et al., 2017). One pilot study with few patients noted that subcutaneous liraglutide prevented the decline of brain glucose consumption, but had no effect on Aβ load or cognition (Gejl et al., 2016). Currently, there are two ongoing larger clinical trials accessing liraglutide neuroprotective effects in AD (NCT01469351, NCT01843075). Other anti-diabetic drugs targeting alternative pathways are also considered to address AD, such as peroxisome-proliferator activated receptor γ (PPAR γ) agonists rosiglitazone and pioglitazone (Miller et al., 2011). Pre-clinical evidence showed that rosiglitazone improved memory and decreased phosphorylated tau (Escribano et al., 2010). However, these studies are also incipient and reported no beneficial effects so far (Harrington et al., 2011; Miller et al., 2011). In the next years, the results of the ongoing larger clinical trials will provide further information about how metabolic status of AD patients interfere on treatment success and also the potential of anti-diabetic therapies in dementia, specially, insulin intranasal use as a possible AD therapy (Femminella et al., 2017).

## Cumulative hypothesis for AD progression

Some puzzles of AD progression are now assembled, however, the sequence of biological alterations that elicits disease development needs clarification. One of the most accepted hypothesis to explain AD is that pathology develops as consequence of the build up of Aβ oligomers and amyloid plaques in the brain (Hardy and Higgins, 1992; Haass and Selkoe, 2007). In familial AD, many mutations are associated with Aβ processing, such as APP and PSEN, supporting this idea. However, it is still elusive the mechanism of Aβ oligomers accumulation and how other mutations apparently non-related to Aβ increase sporadic AD risk (Rao et al., 2014). The understanding that AD can be triggered by inflammation and insulin resistance brought a new perspective. It is possible that allostatic load, multiple environmental effects accumulated during life, accelerates AD pathogenesis. A crosstalk between brain and whole-body metabolic homeostasis is thought to be one of the drivers of sporadic AD (De Felice, 2013; Mattson and Arumugam, 2018).

Environmental factors interfere on cognitive performance even in first years of life, when traumatic brain injury, maternal separation, and psychological trauma might increase the risk of neurodegenerative disease (Barlow, 2005). This correlation is also present in brain injury suffered during adulthood (McKee and Robinson, 2014). Other psychiatric disorders, such as depression, can increase the susceptibility of AD (Ownby et al., 2006). In this context, the brain is subordinate to many types of insults and can also reflect peripheral tissue abnormal function. Sleep disordered breathing is associated with glucose intolerance and insulin resistance that may lead to T2D (Punjabi et al., 2004). Moreover, sleep deprivation also play a role in AD pathogenesis modulating Aβ accumulation (Bliwise, 2004; Kang et al., 2009) and AD-like pathology in mice (Kincheski et al., 2017).

An unhealth lifestyle accelerates the detrimental effects of aging, such as loss of endocrinological control leading to insulin resistance, declines in growth hormone, IGF-1, and sex steroids (Barzilai et al., 2012). A recent study showed that western diet (composed majority by sugar and fat) is associated with hippocampal atrophy (Jacka et al., 2015) and was reported to be associated with a significant rise of AD incidence in Japan (Grant, 2014). Smoking, lack of physical activity, and alcohol abuse are some of other unhealthy practices that increase the risk of AD (Baumgart et al., 2015).

Importantly, older women show faster deterioration of cognition than men, present higher risk for developing AD and abnormal insulin signaling (Li and Singh, 2014; Duarte et al., 2018). Gene network analysis in mice revealed that age-related transcriptional changes happen differently and earlier in female compared to male brain (Zhao et al., 2016). Furthermore, abdominal obesity is correlated with increased death risk in older women and contributes to insulin resistance (Folsom et al., 1993). Besides, other treatments can increase the risk for AD. The Woman Health Initiative observed that hormone replacement therapy combining estrogen plus progestin could upregulate several age related markers, augmenting the risks for cognitive decline, and cardiovascular disease (Writing Group for the Women's Health Initiative Investigators, 2002; Duarte et al., 2018).

In this scenario, it is essential to identify modifiable risks that reduce AD incidence and/or slow down sporadic AD progression. In accordance with the notion that metabolic changes triggers aging, it is possible that AD occurs as a consequence of metabolic dysfunction, that can be caused by cumulative lifelong impacts of lifestyle and other conditions.

## Conclusion

The clinical/epidemiological evidence associating metabolic disorders with dementia has encouraged scientists to search for the biological mechanisms underlying this connection. The pivotal finding that insulin signaling is impaired in AD brains represents a major advance in our current understanding of AD physiopathology. Furthermore, compelling evidence demonstrate that the molecular mechanisms leading to brain insulin resistance in AD share striking similarity to those involved in peripheral insulin resistance in diabetes and obesity. Those include chronic, low-grade inflammation, TNF-α-mediated inhibition of IRS-1, and endoplasmic-reticulum stress. Importantly, these findings also led to the proposition that drugs currently used to overcome peripheral insulin resistance in diabetes may be repurposed to rescue brain insulin signaling in AD. Since insulin signaling is neurotrophic, neuroprotective and plays a key role in synaptic plasticity and cognitive processes, boosting neuronal insulin signaling may come to be a disease modifying therapeutic approach in AD. Indeed, preclinical studies and clinical trials have been performed to test the efficacy of anti-diabetic drugs, including insulin itself, in AD, with some promising results (Table 1). In addition, dissection of the actions of insulin in neuronal and glial cells is likely to foster knowledge on the roles of insulin and related signaling pathways in CNS physiology. Finally, a better comprehension of the crosstalk between peripheral energy metabolism and brain function may also help identifying therapeutic targets and modifiable risk factors for AD and other brain disorders, improving public health policies and public awareness.

## Author contributions

LF, CF, and MV wrote the manuscript. MV and FDF planned and reviewed the final manuscript.

### Conflict of interest statement

The authors declare that the research was conducted in the absence of any commercial or financial relationships that could be construed as a potential conflict of interest.
